# On “Hearing” Voices and “Seeing” Things: Probing Hallucination Predisposition in a Portuguese Nonclinical Sample with the Launay-Slade Hallucination Scale-Revised

**DOI:** 10.3389/fpsyg.2017.01138

**Published:** 2017-07-11

**Authors:** Paula Castiajo, Ana P. Pinheiro

**Affiliations:** ^1^Neuropsychophysiology Lab, CIPsi, School of Psychology, University of Minho Braga, Portugal; ^2^Faculty of Psychology, University of Lisbon Lisbon, Portugal

**Keywords:** nonclinical hallucinations, hallucination predisposition, LSHS, psychometrics proprieties, phenomenology, psychotic risk

## Abstract

The experience of hallucinations is a hallmark of psychotic disorders, but they are also present in other psychiatric and medical conditions, and may be reported in nonclinical individuals. Despite the increased number of studies probing the incidence of nonclinical hallucinations, the underlying phenomenological characteristics are still poorly understood. This study aimed to examine the psychometrics proprieties of the Portuguese adaptation of the 16-item Launay-Slade Hallucinations Scale (LSHS), the phenomenological characteristics of nonclinical hallucinatory experiences in a Portuguese sample, and the relationship between clinical symptoms and hallucination predisposition. Three-hundred-and-fifty-four European Portuguese college students completed the LSHS. Of those, 16 participants with high LSHS scores and 14 with low LSHS scores were further screened for clinical symptoms. A three-factor solution for the LSHS Portuguese version proved to be the most adequate. Intrusive or vivid thoughts and sleep-related hallucinations were the most common. Although, fundamentally perceived as positive experiences, all types of hallucinations were described as uncontrollable and dominating. However, the more pleasant they were perceived, the more controllable they were assessed. In addition, hallucination predisposition was associated with increased clinical symptoms. These results corroborate the lower severity of hallucinations in the general population compared to psychotic individuals. Further, they support an association between clinical symptoms and increased vulnerability to hallucinations. Specifically, increased schizotypal tendencies and negative mood (anxiety and depression) may be related to increased psychotic risk.

## Introduction

Hallucinations represent one of the most intriguing phenomena (e.g., Allen et al., 2008), and have been in the spotlight of researchers from many disciplines for decades. Hallucinatory experiences, usually defined as perceptual experiences that occur in the absence of corresponding external sensory stimulation (Slade and Bentall, 1988), are a clinical manifestation of psychiatric disorders such as schizophrenia (Mueser et al., 1990; Baethge et al., 2005). Even though the experience of hallucinations is considered a hallmark of psychotic disorders (e.g., David, 1994; Johns et al., 2004), hallucinations may also be present in 10–15% of individuals with no clinical diagnosis (e.g., Barrett and Etheridge, 1992; Paulik et al., 2006; Badcock et al., 2008; Sommer et al., 2010). These findings provided support for a continuum model of psychotic experiences that extends not only across diagnostic categories, but also into the (non-clinical) general population (e.g., Johns and van Os, 2001; Bradbury et al., 2009; Brébion et al., 2010). Three distinct states are thought to represent the phenotypic continuum of psychosis: (1) brief and attenuated psychotic experiences in the healthy population (typically hallucinations and delusions) observed in the least severe extreme; (2) persistent sub-clinical psychotic symptoms without functional impairment occurring from the least severe to the most severe extreme; and (3) psychotic disorders with symptoms that cause clinically significant distress and functional impairment occurring in the most severe extreme (van Os et al., 2009). Of note, nonclinical cases represent the largest proportion of the full continuum (van Os et al., 2009). Differences between clinical and nonclinical psychotic symptoms seem to be quantitative rather than qualitative (e.g., Larøi and van der Linden, 2005a; Larøi, 2012). Indeed, the onset of psychosis is often preceded by an increased frequency of nonclinical psychotic symptoms (e.g., Hanssen et al., 2005; Larøi and van der Linden, 2005a; Dominguez et al., 2011; Larøi, 2012) in co-occurrence with clinical conditions conditions such as anxiety, depressed mood, suspiciousness, disorganization, irritability, social withdrawal, poor functioning, and cognitive and behavioral changes (e.g., Yung and McGorry, 1996; Yung et al., 2003). The occurrence of nonclinical hallucinations represents a risk factor for conversion to full psychosis (e.g., Kelleher and Cannon, 2011), even though it is not necessarily followed by a psychotic diagnosis (Verdoux et al., 1999; Johns and van Os, 2001).

Hallucinations may occur in different sensory modalities (e.g., auditory, visual, olfactory, tactile, and gustatory). Nonetheless, auditory hallucinations are the most common, especially in patients with schizophrenia (David and Busatto, 1998). They are often perceived as “voices” talking to each other (auditory verbal hallucinations—AVHs; Mueser et al., 1990; Nayani and David, 1996). AVHs have been consistently reported in 70% of schizophrenia patients (e.g., David, 1994; Johns et al., 2004), as well as in other clinical disorders such as affective psychosis, depression, bipolar, or posttraumatic stress disorder (e.g., Asaad, 1990; Tien, 1991). However, the experience of “hearing voices” may also occur in 5–28% of the normal population (e.g., Tien, 1991; Johns et al., 2004; de Leede-Smith and Barkus, 2013). Nonetheless, there are important (e.g., phenomenological) differences between clinical and nonclinical AVHs (e.g., Choong et al., 2007; Badcock et al., 2008; Brébion et al., 2010; Larøi et al., 2012; de Leede-Smith and Barkus, 2013). Particularly, AVHs occur with increased frequency and duration in psychotic compared to nonpsychotic individuals (de Leede-Smith and Barkus, 2013). Moreover, AVHs are perceived as more uncontrollable and unpleasant in psychotic individuals (de Leede-Smith and Barkus, 2013). The loudness (a little softer than the self-voice), attribution (external source), and location of the hallucinated voice (inside the head) are the main similarities shared by clinical and nonclinical individuals (de Leede-Smith and Barkus, 2013).

Visual hallucinations (VHs) are the second most common type of hallucinations in psychotic patients (prevalence of 24–72%; Cummings and Miller, 1987), even though their prevalence might be underestimated (Thomas et al., 2007). VHs may also occur in a wide range of clinical conditions, such as ophthalmologic diseases, neurologic disorders, toxic and metabolic disorders, and psychiatric disorders (e.g., schizophrenia, affective psychosis and bipolar disorder; Cummings and Miller, 1987; Waters et al., 2014), as well as in nonclinical individuals (Tien, 1991; Johns and van Os, 2001). Although, visual hallucinations are much less common in nonclinical compared to clinical samples, they seem to be more prevalent than auditory hallucinations in healthy individuals (Tien, 1991; Ohayon, 2000).

Whereas most of the studies have focused on auditory and visual forms of hallucinations (e.g., Cummings and Miller, 1987; Stephane et al., 2003; Sanjuan et al., 2004; Langdon et al., 2009; Teeple et al., 2009; McCarthy-Jones et al., 2014), fewer studies examined hallucinations in other sensory modalities (e.g., Mueser et al., 1990; Thomas et al., 2007; Lewandowski et al., 2009). Despite their rare frequency, olfactory, gustatory, and tactile hallucinations have been additionally reported in both psychotic (Lewandowski et al., 2009) and nonclinical samples (Tien, 1991). Nonetheless, auditory and visual forms represent the major risk factors for a psychiatric diagnosis (Ohayon, 2000; de Leede-Smith and Barkus, 2013).

In the last decades, a growing number of studies probed the pathophysiological mechanisms underlying the experience of hallucinations in nonclinical samples (e.g., Posey and Losch, 1983; Young et al., 1986; Barrett and Etheridge, 1992; Paulik et al., 2007; Vercammen and Aleman, 2010). While it is important to consider that nonclinical hallucinations are typically less severe than clinical hallucinations (e.g., de Leede-Smith and Barkus, 2013), the study of this phenomenon in a nonclinical population is particularly advantageous as it avoids confounding effects of medication and hospitalization that are often ascribed to clinical samples (e.g., Kühn and Gallinat, 2010).

The existing studies have most commonly selected participants from university settings (e.g., Morrison et al., 2000; Larøi and van der Linden, 2005b; Paulik et al., 2006; Fonseca-Pedrero et al., 2010). In this context, the Launay-Slade Hallucination Scale (LSHS; Launay and Slade, 1981), and its subsequent revised versions (e.g., Bentall and Slade, 1985; Morrison et al., 2000, 2002; Larøi and van der Linden, 2005b), has been the most commonly used instrument to probe hallucination predisposition. Of note, nonclinical individuals with higher LSHS scores were found to share phenomenological, cognitive, neuropsychological, and psychophysiological similarities with psychotic patients with hallucinations (Bentall et al., 1989; de Leede-Smith and Barkus, 2013). The LSHS original English version (Launay and Slade, 1981) consists of 12 items in a true or false response format. Items were subsequently rephrased by replacing negatively formulated content with positive content in the first modified version of the scale (Bentall and Slade, 1985). Aiming to increase response variability, Bentall and Slade (1985) also replaced the true-or-false response format with a 5-point Likert scale. As previous versions of the LSHS did not address visual hallucinatory experiences, subsequent versions by Morrison et al. (2000, 2002) have incorporated additional items that tap into visual forms of hallucinations (Morrison et al., 2000, 2002). These studies have also removed the “unsure” option with the aim of preventing the tendency to choose the midpoint option of the scale (Morrison et al., 2000, 2002). Due to the lack of items probing the experience of hallucinations other than in the auditory and visual modalities, two Belgian versions of the LSHS, including 17 and 16 items, respectively, were developed (Larøi et al., 2004; Larøi and van der Linden, 2005b). Compared to the LSHS first Belgian version (Larøi et al., 2004), the subsequent Belgian version (Larøi and van der Linden, 2005b) has only two modifications: an item was excluded due to its extremely low response rating (“In the past I have heard the voice of God or one of his messengers speaking to me”); and the item “Sometimes, when I look at things such as chairs and tables, they are unreal or strange” was replaced with “Sometimes I have seen things or animals when nothing was in fact there.” This replacement aimed to provide a more appropriate visual hallucination item, considering the lack of this type of items in the LSHS version of Morrison et al. (2000). From all the LSHS adaptations, the two versions developed by Larøi et al. (2004), Larøi and van der Linden (2005b) are the most complete, as they include items that tap into distinct forms of hallucinations (auditory, visual, olfactory, tactile, hypnagogic, and hypnopompic), and as those items are further assessed in other relevant dimensions (e.g., frequency, duration, degree of control, and affective content).

Although, an exploratory factor analysis of the LSHS has shown that hallucination predisposition is better represented by a multi-factor structure (e.g., Waters et al., 2003; Larøi et al., 2004; Paulik et al., 2006), the number and type of dimensions that characterize this predisposition are not consensual: two-factor (e.g., Morrison et al., 2000; Serper et al., 2005; Fonseca-Pedrero et al., 2010), three-factor (e.g., Aleman et al., 2001; Waters et al., 2003; Paulik et al., 2006), four-factor (e.g., Levitan et al., 1996; Larøi et al., 2004; Cangas et al., 2009; Vellante et al., 2012), and five-factor (e.g., Larøi and van der Linden, 2005b) dimensions were proposed. Whereas some of these inconsistencies may be accounted for by differences in LSHS versions or response formats, and by differences in the samples (clinical and nonclinical) examined, study discrepancies were reported even when using the same LSHS version. For example, using the LSHS developed by Bentall and Slade (1985), Serper et al. (2005) found a two-factor solution (“subclinical factor” and “clinical factor”), whereas Aleman et al. (2001) and Waters et al. (2003) obtained a three-factor solution. These two later three-factor solutions also differed from each other (“tendency toward hallucinatory experiences,” “subjective externality of thought,” and “vivid daydreams”; “vivid mental events,” “hallucinations with a religious theme,” and “auditory and visual perceptual hallucinatory experiences,” respectively). Moreover, whereas a five-factor solution was found in the LSHS adaptation of Larøi and van der Linden (2005b), a four-factor structure was proposed by the Italian adaptation of the Belgian version (Vellante et al., 2012). Despite the controversy surrounding this topic, there is substantial evidence that all LSHS versions are especially reliable, having adequate psychometric properties (e.g., Waters et al., 2003; Cella et al., 2008) and temporal stability (e.g., Morrison et al., 2002). Moreover, the LSHS is a versatile instrument that can be used to measure hallucination predisposition in both nonclinical (e.g., Morrison et al., 2000; Waters et al., 2003; Larøi et al., 2004; Larøi and van der Linden, 2005b) and clinical individuals (e.g., Levitan et al., 1996; Serper et al., 2005). As such, it was adapted to many languages, including Dutch (Aleman et al., 2001), Spanish (Fonseca-Pedrero et al., 2010), French (Larøi et al., 2004; Larøi and van der Linden, 2005b), and Italian (Vellante et al., 2012). The validation and adaptation of the LSHS in different languages represent a cross-cultural validation of the hallucination predisposition construct (Hui and Triandis, 1985).

Note that the adaptation of a scale or survey to a specific language and cultural context requires specific statistical analyses that determine whether the instrument has adequate measurement properties to be used not only in the target population, but also in cross-cultural studies. Three important indices are considered in the evaluation of the quality of an assessment instrument: (1) sensitivity (i.e., the ability to discriminate between individuals—discrimination, and to detect individual changes over time—responsiveness to change; Kirshner and Guyatt, 1985), (2) validity (i.e., the ability to capture the construct under study—construct validity, and to produce reliable empirical knowledge—internal validity; Kimberlin and Winterstein, 2008), as well as (3) consistency and reliability (i.e., the ability to consistently produce the same result if the instrument is administered again—stability, and to achieve agreement between items—internal consistency; Kimberlin and Winterstein, 2008).

### Aims and hypotheses of the current study

The current study aimed to validate and adapt the LSHS version developed by Larøi and van der Linden (2005b) for the Portuguese population, by examining its psychometric proprieties (sensitivity, internal validity, and internal consistency). In addition, the psychometric results of the Portuguese LSHS adaptation were compared with the Belgian LSHS original validation. The decision to use this LSHS version was motivated by the fact that it comprises items concerning several types of hallucinatory experiences (auditory, visual, olfactory, and tactile), and a response format that allows measuring additional relevant aspects of these experiences (e.g., prevalence, frequency, degree of control, affective content of the hallucinatory experience). We expected to find a five-factor structure in the Portuguese adaptation of the LSHS, similarly to Larøi and van der Linden (2005b).

In a second step, we sought not only to describe the phenomenological characteristics of nonclinical Portuguese hallucinatory experiences (e.g., prevalence, frequency, degree of control, affective content of the hallucinatory experience), but also to examine the relationship between them (Larøi and van der Linden, 2005b). Differences between males and females, which were not tested in the previous adaptations of the scale, were also examined. The analysis of sex differences was motivated by previous evidence demonstrating that women are more commonly affected by nonclinical hallucinations when compared to men (Young et al., 1986; Tien, 1991; Maric et al., 2003; van Os, 2003). Consequently, we hypothesized that nonclinical hallucinations would be more prevalent in female relative to male participants. Moreover, based on previous findings (Johns et al., 2002; Larøi and van der Linden, 2005b), we predicted that unpleasant experiences would be related to attributions of lower controllability over the end and reappearance of these experiences, as well as with higher frequency ratings.

Following Larøi and van der Linden (2005b), the third aim of the current study was to clarify whether the hallucinatory experiences were related to the use of alcohol or drugs. In addition, we aimed to determine the association between LSHS ratings and anxious-depressive symptomatology and schizotypal tendencies. These later measures were not included in the study of Larøi and van der Linden (2005b). Previous studies have shown that the presence of both anxious-depressive symptomatology and schizotypal tendencies (Paulik et al., 2006; Smith et al., 2006; Barkus et al., 2007; de Leede-Smith and Barkus, 2013) might indicate increased hallucination predisposition. Therefore, we hypothesized that the prevalence of hallucinatory experiences would be higher in individuals reporting more clinical symptoms.

## Methods

### Participants

A total sample of 354 European Portuguese college students (mean age = 24.13, *SD* = 6.61 years, ranging from 18 to 64 years; 266 females) participated in the study. Participants were recruited, via email, from several universities located in the north, center and south of Portugal, following a sampling procedure commonly used in prior studies with college students (e.g., Jones and Fernyhough, 2009; Richardson and Garavan, 2009; Stainsby and Lovell, 2014). The education level varied from 12 to 21 years (*M* = 14.52, *SD* = 1.83). Data from the 354 participants who responded to the questionnaire were all included in the data analyses. At the time participants answered the questionnaire, 328 were students, whereas 26 had recently finished their undergraduate or graduate studies. Considering the nature of this recruitment method, a specific response rate could not be determined.

Of those, 16 participants (mean age = 23.50, *SD* = 8.53 years, ranging from 18 to 53 years; 11 females) with high LSHS scores (mean score = 35.13, *SD* = 8.03, ranging from 25 to 51 points), and 14 participants mean age = 21.40, *SD* = 5.24 years, ranging from 18 to 37 years; 13 females) with low LSHS scores (mean score = 14.57, *SD* = 4.69, ranging from 7 to 20 points) were further screened for clinical symptoms.

The study protocol was approved by the local ethics committee (‘Subcomissão de Ética para as Ciências da Vida e da Saúde’—CECVS—064/2014, from the University of Minho). Informed written consent was obtained for each participant prior to their involvement in the study.

### Materials

The Portuguese adaptation of the Belgian modified version of the LSHS (Larøi and van der Linden, 2005b, originally developed by Launay and Slade, 1981) was used to assess hallucination predisposition in college students.

The translation and adaptation of the modified version of the LSHS (Larøi and van der Linden, 2005b) for the European Portuguese language was carried out using the back-translation procedure. This procedure involves three independent steps. First, the original Belgian version (Larøi and van der Linden, 2005b who translated the scale from French into English) was translated into European Portuguese by a native speaker who was highly proficient in the use of the English language. Subsequently, the European Portuguese version was back translated into the original English language by three other subjects who were blind to the original version. Finally, all the translations as well as the original version were reviewed to verify semantic, idiomatic, experiential and conceptual equivalence, and after discussing any potential discrepancies, a consensus was reached regarding the final version of the Portuguese LSHS.

The LSHS current version includes 16 items tapping into different forms of hallucinations (auditory, visual, olfactory, tactile, hypnagogic, and hypnopompic), which are measured using a 5-point Likert scale, from 0 to 4 (0 = “*definitely does not apply to me*,” 1 = “*possibly does not apply to me*,” 2 = “*unsure*,” 3 = “*possibly applies to me*,” and 4 = “*definitely applies to me*”). The total score ranges between 0 and 64, with higher scores indicating higher hallucination predisposition. Consistent with the first Belgian LSHS version (Larøi et al., 2004), this version includes five additional items that were not part of the LSHS short versions (Launay and Slade, 1981; Bentall and Slade, 1985; Morrison et al., 2000, 2002): an item related to olfactory hallucinatory experiences (“In the past, I have smelled a particular odor when there was nothing there”), an item related to tactile hallucinatory experiences (“I have had the feeling of touching something or being touched and then found that nothing or nobody was there”), an item related to the experience of feeling the presence of someone close who has already died (“On certain occasions I have had the feeling of the presence of someone close who has deceased”), an item which is simultaneously related to visual, auditory and tactile sensory experiences (“Sometimes, immediately prior to falling asleep or upon awakening, I have had the experience of having seen or felt or heard something or someone that wasn't there or the feeling of being touched even though no one was there”), and an item that addresses sensations of flying and floating, and out-of-body experiences (“Sometimes, immediately prior to falling asleep or upon awakening, I have had a sensation of floating or falling or that I left my body temporarily”). Responses such as “*possibly applies to me”* or “*definitely applies to me”* (3 or 4 points) were subsequently rated on a 5-point Likert scale measuring frequency of occurrence (from 0 = “*it occurs very rarely”* to 4 = “*it occurs very often”*), degree of control (over the end of the experience, varying from 0 = “*it's very easy to cease the experience”* to 4 = “*it's very difficult to cease the experience”;* and over the beginning of the experience, varying from 0 = “*it's very easy to avoid the experience”* to 4 = “*it's very difficult to avoid the experience,”* and affective content of the experience (from 0 = “*the experience is very negative”* to 4 = “*the experience is very positive”*). Moreover, participants who responded “*possibly applies”* or “*definitely applies”* (3 or 4 points) to at least one item were further invited to complete five complementary items on a true-or-false response format. These questions aimed to clarify whether the hallucinatory experiences concerned them personally or not, whether these experiences involved other close persons and past events, and if the hallucinatory experiences occurred during stressful events or under the influence of drugs or alcohol. The Portuguese translation of the LSHS items is presented in Appendix A.

In order to collect additional relevant information, hallucination predisposition items were interspersed with several fillers items: seven items concerning psychopathology and schizotypal tendencies (e.g., “I feel more upset or angry than usual,” “I am rarely sad or depressed,” “I describe myself as an anxious person,” “People usually describe me as an odd person”), and 10 items selected from the Marlow–Crowne Social Desirability Scale (Crowne and Marlow, 1960; e.g., “I am always friendly, even with people who are unpleasant,” “Sometimes, I feel resentful for not having things the way I like”). Clinical filler items were based on questionnaires commonly used in clinical assessments such as the Brief Symptom Inventory (BSI, Derogatis and Spencer, 1982) and the Schizotypal Personality Questionnaire (SPQ; Raine, 1991). All filler items were randomly distributed through the LSHS questionnaire, and responses were provided by using the same scoring scale (5-point Likert scale).

The BSI and the SPQ were used to assess clinical symptoms in both high and low LSHS participants, in a second stage of the study. The Portuguese adaptation of the BSI (Derogatis and Spencer, 1982; adapted by Canavarro, 1999) was used to examine the presence of psychological distress and psychiatric disorders. The BSI is a self-report questionnaire with 53 items distributed across nine subscales (Somatization, Obsessive-Compulsive, Interpersonal Sensitivity, Depression, Anxiety, Hostility, Phobic Anxiety, Paranoid Ideation, and Psychoticism) and three additional scales (Global Severity Index, Positive Symptoms Total, and Positive Symptoms Distress Index) that capture global psychological distress. Items are evaluated on a 5-point Likert scale, ranging from 0 (“not at all”) to 4 (“extremely”). Total scores range between 0 and 24 for three subscales (Obsessive-Compulsive, Depression, and Anxiety), 0 and 20 for four subscales (Hostility, Phobic Anxiety, Paranoid Ideation, and Psychoticism), 0 and 16 for one subscale (Interpersonal Sensitivity), and 0 and 28 for one subscale (Somatization). Moreover, scores ≥1.7 in the Positive Symptoms Distress Index (PSDI) may indicate emotional distress.

The Portuguese adaptation of the SPQ (Raine, 1991, adapted by Santos, 2011) was used to assess schizotypal traits. The 74-item self-rated SPQ comprises three factors (Cognitive-Perceptual, Interpersonal, and Disorganized). These factors in turn include distinct subscales: the Cognitive-Perceptual (Ideas of Reference, Odd Beliefs, Unusual Perceptual Experiences, and Suspiciousness), and Interpersonal (Excessive Social Anxiety, No Close Friends, Constricted Affect, and Suspiciousness) factors include four subscales, whereas the Disorganized factor includes two subscales (Odd or Eccentric Behavior and Odd Speech). Items are rated using a dichotomous response format (“yes,” “no”). Total scores range between 0 and 74: the higher the number of “yes” responses, the more severe the schizotypal traits.

### Procedure

Data were collected from April 2014 to February 2015. College students were invited via email to participate in a study in which they were asked to respond to a questionnaire about perceptual experiences, by accessing a *website* in a HTLM format, specifically developed for that purpose. To encourage survey participation, a voucher was drawn. The online survey began by introducing the goal of the study and providing specific instructions about the questionnaire. Prior to the presentation of the questionnaire, participants were reminded about confidentiality and rights. Those who were interested in participating in the study ensured their participation by providing online informed consent. After providing socio-demographic information (age, sex, education level), participants were asked to complete the questionnaire. During the presentation of the questions, items remained at the center of the computer screen until a response was made. Answers were automatically saved after the participants' selection using the mouse. There was no time limit to complete the questionnaire.

Participants were subsequently assigned to one of two groups on the basis of hallucination predisposition by a median split. Those with high LSHS scores (≥20 points) and those with low LSHS scores (≤20 points), and who agreed to enroll in a second stage of the study, were asked to complete the BSI and the SPQ in a face-to-face assessment session. To encourage participation, vouchers or course credits were provided.

### Data analyses

Statistical analyses were performed using the IBM SPSS Statistics 22.00 (SPSS, Corp., USA) software package. First, psychometric properties of the LSHS Portuguese adaptation were examined by assessing its sensitivity, internal validity and internal consistency, and reliability. A frequency analysis for each item was conducted to determine whether all response categories were represented in this sample (*sensitivity*). The factor structure of the questionnaire was performed through Principal Component Analysis using Varimax rotation (*internal validity*). We confirmed whether data were based on the Kaiser-Meyer-Olkin (KMO) criteria and Bartlet's test of sphericity. Only factors with eigenvalues greater than 1 were retained. Subsequently, three Principal Component Analysis were conducted to determine the best solution for the Portuguese version of the questionnaire: (1) an unforced factor analysis with a cut-off point of 0.30 for factor loadings; (2) a forced five-factor solution with factor loadings above 0.50, as suggested in the original factor analysis (Larøi and van der Linden, 2005b); and (3) a forced analysis with Promax rotation in order to extract four factors loading >0.30, as proposed in a subsequent Italian adaptation (Vellante et al., 2012) of the revised scale presented by Larøi and van der Linden (2005b). For the forced five- and four-factor solutions, items loading above the established saturation values (0.50 and 0.30, respectively) on more than one factor were retained in the same factors of the previous studies. The reliability was quantified by measuring the internal consistency of the questionnaire with item-total score correlations (correlation of each item with the total of the remaining items), as well as with Cronbach's alpha coefficients (*internal consistency and reliability*). The Cronbach's alpha coefficient was calculated for the total score, as well as for each factor, and values ≥0.70 were considered acceptable. Items were considered to have adequate consistency if their item-total correlation fell between 0.275 and 0.75 (Pedhazur and Schmelkin, 1991).

Second, descriptive analyses were conducted to describe the phenomenological characteristics of the hallucinatory experiences (prevalence, frequency of occurrence, perceived degree of control, and affective content of the hallucinatory experience for each factor and for each item) and other specific aspects related to them (whether the hallucinatory experiences concerned participants personally, if they involved relatives or friends, events already experienced and stressful events or difficulties, as well as if they occurred under the influence of drugs or alcohol).

Third, an exploratory data analysis was conducted using the Shapiro–Wilk test, the Kolmogorov–Smirnov test, and measures of skewness and kurtosis. This analysis revealed that data for some of the variables under study (prevalence, frequency, valence, and control) were not normally distributed. Nonetheless, a direct comparison of results between nonparametric and parametric tests revealed identical results. In such a case, it has been suggested to report the results from parametric tests as they have more statistical power than nonparametric tests (Fife-Schaw, 2006). Therefore, parametric tests were selected as no discrepancies were observed between parametric and nonparametric test results, and considering prior simulation studies (Elliott and Woodward, 2007; Ghasemi and Zahediasl, 2012) that indicate that parametric tests perform well with non-normal distributions if the sample size is large enough (*n* > 30). Hence, differences between males and females in hallucinatory experiences were examined using independent samples *t*-tests, whereas Pearson correlation coefficients were used to probe the relationship between the affective content of the hallucinatory experiences and the perceived degree of control over the experiences (means for the items tapping into the perceived control over the beginning vs. end of the experiences were calculated separately), as well as between the affective content of the hallucinatory experiences and their frequency of occurrence.

Finally, independent samples *t*-tests were used to probe differences between individuals with high vs. low hallucination predisposition in clinical symptomatology. Additionally, the relationship between LSHS and clinical scores was assessed using Pearson correlation coefficients. Parametric statistical tests were used as the normality assumption was verified.

## Results

### Psychometric properties of the portuguese adaptation of the LSHS

#### Sensitivity

The Portuguese version of the LSHS was found to have appropriate sensitivity as all items were successful in discriminating between the five response categories. Nonetheless, whereas participants tended to select higher values (3 and 4 points) in five of the items (1, 2, 3, 7, and 12) and unsure values (2 points) in one of the items (5), 10 items (4, 6, 8, 9, 10, 11, 13, 14, 15, and 16) were more likely to receive lower values (0 points).

#### Internal validity

In the first Principal Component Analysis (unforced analysis) a three-factor structure explaining 53% of the total variance was found. The first factor yielded the highest proportion of explained variance (24.13%), and included 7 items. The second factor accounted for additional 17.40% of the total variance and was composed of 3 items, whereas the third factor, explaining 11.47% of the variance, included 6 items. For this factorial solution, the Bartlet's test of sphericity was statistically significant (*p* < 0.001), and the Kaiser-Meyer-Olkin (KMO) index was close to 1 (0.88), which reflects goodness-of-fit of the factor analysis. Eigenvalues for the three factors were all >1. Items that loaded above 0.30 on more than one factor were retained in the factor that included other similar items. Table 1 displays the factor analysis of the Portuguese version of the LSHS, which resulted in a three-factor structure.

**Table 1:** Three-factor structure of the Portuguese adaptation of the LSHS.

**Item**	**Factor**		
	**1**	**2**	**3**
4. In the past, I have had the experience of hearing a person's voice and then found that no one was there.	**0.67**		
8. I often hear a voice speaking my thoughts aloud.	**0.34**	0.55	
9. I have been troubled by hearing voices in my head.	**0.67**		
10. On certain occasions, I have seen the face of a person in front of me, but there was no one.	**0.73**		
14. In the past, I have smelt a particular odor when there was nothing there.	**0.55**		
15. I have had the feeling of touching something or being touched and then found that nothing or no one was there.	**0.79**		
16. Sometimes I have seen things or animals when nothing was in fact there.	**0.76**		
5. The sounds I hear in my daydreams are generally clear and distinct.		**0.78**	
6. The people in my daydreams seem so true to life that I sometimes think that they are.		**0.73**	
7. In my daydreams I can hear the sound of a tune almost as clearly as if I were actually listening to it.		**0.73**	
1. Sometimes a passing thought will seem so real that it frightens me.		0.50	**0.44**
2. Sometimes my thoughts seem as real as actual events in my life.		0.51	**0.55**
3. No matter how hard I try to concentrate on my work unrelated thoughts always creep into my mind.			**0.74**
11. Sometimes, immediately prior to falling asleep or upon awakening, I have had the experience of having seen or felt or heard something or someone that wasn't there or the feeling of being touched even though no one was there.	0.64		**0.38**
12. Sometimes, immediately prior to falling asleep or upon awakening, I have had a sensation of floating or falling or that I left my body temporarily.			**0.56**
13. On certain occasions I have had the feeling of the presence of someone close who has deceased.	0.46		**0.37**
Eigenvalue	5.58	1.78	1.11
Explained variance (%)	24.13	17.40	11.47
Cronbach α	0.82	0.74	0.71

*Primary loading values (>0.30) are presented in bold; secondary loadings are underlined*.

However, this three-factor structure was not in accordance with the five-factor structure proposed by the authors of the original questionnaire (Factor I: “Sleep-related hallucinations,” Factor II: “Vividness of daydreams,” Factor III: “Intrusive or vivid thoughts,” Factor IV: “Auditory hallucinations,” and Factor V: “Visual hallucinations”). To extract the same number of factors, a second factor analysis was conducted. Even though the forced five-factor solution accounted for a greater amount of the total variance (64.35%) than the unforced three-factor solution (53%), the fourth and the fifth factors obtained eigenvalues below 1 (0.95 and 0.87, respectively). Moreover, three items loaded below 0.50 (1, 8, and 15) in the current five-factor solution, and therefore were excluded. Factor I explained 19.22% of the variance and included four items; Factor II accounted for 15.75% of the variance and included three items; Factor III, explaining 10.15% of the total variance, retained two items; Factor IV accounted for 9.90% of the variance and retained two items; and Factor V explained the lowest proportion of variance (9.32%), and included two items. Whenever possible, items loading above 0.50 on more than one factor were included in the same factor of the original structure. Retained items and corresponding factor loadings, as well as comparative results between this study and the original study (Larøi and van der Linden, 2005b), are presented in Table 2.

**Table 2:** Five-factor structure of the Portuguese adaptation of the LSHS and the Belgian original five-factor structure.

**Item**	**Portuguese five-factor**					**Belgian five-factor**				
	**1**	**2**	**3**	**4**	**5**	**1**	**2**	**3**	**4**	**5**
4.	0.72								0.68	
8.	0.43^a^								0.63	
9.	0.72								0.73	
10.	0.76									0.71
16.	0.67									0.65
5.		0.79					0.73			
6.		0.66					0.69			
7.		0.79					0.79			
11.			0.70			0.71				
12.			0.67			0.72				
15.			0.45^a^			0.73				
13.				0.71		0.58				
14.				0.62						
1.					0.42^a^			0.64		
2.					0.61			0.70		
3.					0.80			0.65		
Eigenvalue	5.58	1.78	1.11	0.95^b^	0.87^b^	5.03	1.96	1.83	1.61	1.46
Variance %	19.22	15.75	10.15	9.90	9.32	29.00	10.00	9.00	7.00	4.00
Cronbach α	0.79	0.74	0.58^c^	0.49^c^	0.50^c^	0.74	0.72	0.68	0.76	—

a *Items loading <0.50 were excluded. This occurred for three items (1, 8, and 15)*.

b *Eigenvalues below 1*.

c *Cronbach α values below 0.70*.

Following the Italian validation of the revised LSHS version (Vellante et al., 2012), a final forced Principal Components Analysis was conducted to extract four factors. The four-factor solution for the Portuguese adaptation of the questionnaire proposed by Vellante et al. (2012) explained 58.92% of the total variance. The percentage of variance explained by each factor was 34.90% for Factor I (3 items), 11.14% for Factor II (4 items), 6.96% for Factor III (6 items), and 5.92% for Factor IV (3 items). All items saturated above 0.30 in the same factors of the Italian study. However, only the first three factors showed eigenvalues >1. Factor loadings and comparative results between the Portuguese study and the Italian study are listed in Table 3.

**Table 3:** Four-factor structure of the Portuguese adaptation of the LSHS and the Italian four-factor structure of the Belgian LSHS version.

**Item**	**Portuguese four-factor**				**Italian four-factor**			
	**1**	**2**	**3**	**4**	**1**	**2**	**3**	**4**
8.	0.47				0.51			
9.	0.76				0.64			
10.	0.81				0.94			
4.		0.33						0.31
5.		0.82						0.75
6.		0.73						0.83
7.		0.80						0.77
11.			0.78			0.75		
12.			0.66			0.67		
13.			0.46			0.68		
14.			0.66			0.59		
15.			0.73			0.76		
16.			0.80			0.39		
1.				0.67			0.74	
2.				0.77			0.88	
3.				0.74			0.62	
Eigenvalue	5.58	1.78	1.11	0.95^a^	6.71	1.82	1.22	0.89
Variance %	34.90	11.14	6.96	5.92	41.90	11.50	7.60	5.60
Cronbach α	0.62^b^	0.71	0.77	0.64^b^	0.85	0.85	0.83	0.86

a *Eigenvalues below 1*.

b *Cronbach α values below 0.70*.

Based on the three Principal Components Analysis, a three-factor solution proved to be the most adequate. Therefore, the Portuguese version of the LSHS includes a total of 16 items, distributed by the 3 factors that emerged from the analysis. Factor I was named “Auditory, visual, olfactory, and tactile hallucinations” as it encompasses the seven items related to the four types of hallucinations; Factor II was named “Vividness of daydreams” and comprises the three same items of the original version; and Factor III was named “Intrusive or vivid thoughts and Sleep-related hallucinations.” This last factor comprises six items that were distributed by two independent factors in the original version (“Intrusive or vivid thoughts” and “Sleep-related hallucinations”). Even though the items were regrouped into 3 factors in the Portuguese version of the LSHS only, the 15 items of the original five-factor version were kept. Moreover, item 14 (concerning the olfactory sensory modality) was retained in the current version, although it was excluded from the Belgian study as its saturation value was not within the acceptable interval (>0.50). The following analyses were only run on the final three-factor structure of the Portuguese LSHS.

#### Internal consistency and reliability

A Cronbach's alpha of 0.87 was found for the global score. Table 1 shows Cronbach's alpha values for each factor of the Portuguese version of the LSHS, with values ranging between a minimum of 0.71 for Factor III and a maximum of 0.82 for Factor I. These values indicate a high internal consistency and reliability. Moreover, an item-total correlation above 0.30 was found for all items (0.31–0.62 range), revealing a high item-total consistency (see Table 4). Taken together, these results confirm the internal consistency of the Portuguese adaptation of the LSHS.

**Table 4:** Mean scores, standard deviations, item-total correlations, and alpha levels after item elimination from the LSHS Portuguese adaptation (*n* = 354).

**Item**	**Mean**	**SD**	**Item-total correlation**	**Alpha if item was deleted**
1. Sometimes a passing thought will seem so real that it frightens me.	1.95	1.30	0.50	0.86
2. Sometimes my thoughts seem as real as actual events in my life.	2.16	1.27	0.55	0.86
3. No matter how hard I try to concentrate on my work unrelated thoughts always creep into my mind.	2.63	1.14	0.31	0.87
4. In the past, I have had the experience of hearing a person's voice and then found that no one was there.	0.93	1.26	0.54	0.86
5. The sounds I hear in my daydreams are generally clear and distinct.	1.64	1.19	0.50	0.86
6. The people in my daydreams seem so true to life that I sometimes think that they are.	1.22	1.20	0.54	0.86
7. In my daydreams I can hear the sound of a tune almost as clearly as if I were actually listening to it.	1.64	1.36	0.47	0.86
8. I often hear a voice speaking my thoughts aloud.	1.95	1.30	0.49	0.86
9. I have been troubled by hearing voices in my head.	0.49	0.90	0.49	0.86
10. On certain occasions, I have seen the face of a person in front of me, but there was no one.	0.45	0.89	0.59	0.86
11. Sometimes, immediately prior to falling asleep or upon awakening, I have had the experience of having seen or felt or heard something or someone that wasn't there or the feeling of being touched even though no one was there.	1.14	1.32	0.55	0.86
12. Sometimes, immediately prior to falling asleep or upon awakening, I have had a sensation of floating or falling or that I left my body temporarily.	2.16	1.51	0.45	0.86
13. On certain occasions I have had the feeling of the presence of someone close who has deceased.	0.84	1.19	0.45	0.86
14. In the past, I have smelt a particular odor when there was nothing there.	0.78	1.13	0.48	0.86
15. I have had the feeling of touching something or being touched and then found that nothing or no one was there.	0.73	1.09	0.58	0.86
16. Sometimes I have seen things or animals when nothing was in fact there.	0.55	0.93	0.62	0.86

### Phenomenological features of the hallucinatory experiences

#### Prevalence, frequency of occurrence, perceived degree of control, and affective content

Hallucinatory experiences were reported by 10% of the sample (the cut-off point at the upper 90th percentile was 35 points). Table 5 shows the prevalence, frequency of occurrence, perceived degree of control, and affective content of the hallucinatory experiences for each of the three LSHS factors. Factor III (Intrusive or vivid thoughts and Sleep-related hallucinations) received the highest prevalence and frequency of occurrence ratings (42 and 33%, respectively), followed by Factor II (Vividness of daydreams; 26 and 21%, respectively), and Factor I (Auditory, visual, olfactory and tactile hallucinations; 10 and 8%, respectively). In contrast, few participants described their vivid hallucinatory experiences as very rare (2% for Factor III, 1% for Factor I, and 0.6% for Factor II). Regarding the perceived degree of control, 39% (Factor III), 24% (Factor II), and 10% (Factor I) of participants rated these experiences as being poorly controllable, while very few considered them as being highly controllable (1, 0.7, and 0.5%, respectively). Finally, hallucinatory experiences were described as more pleasant than unpleasant: Factor III experiences were characterized as the most pleasant (35%), followed by those included in Factor II (24%) and Factor I (9%), whereas only 2% (Factor III) and 0.3% (Factor II and Factor I) of participants associated their experiences with unpleasant emotions.

**Table 5:** Overall number of participants and (percentages) for each LSHS factor according to prevalence, frequency of occurrence, perceived degree of control, and affective content.

**Factor**	**Prevalence^a^**	**Frequency^b^ (rare-often)**	**Control^c^ (low-high)**	**Affective content^d^ (negative-positive)**
I.	36 (10%)	5–28 (1–8%)	34–2 (10–0.5%)	2–32 (0.5–9%)
II.	92 (26%)	2–76 (0.6–21%)	84–3 (24–0.7%)	1–86 (0.3–24%)
III.	148 (42%)	9–116 (2–33%)	137–5 (39–1%)	7–126 (2–35%)

a *Percentage of participants who answered “possibly applies to me” or “definitely applies to me” (3 or 4 points)*.

b *Percentage of participants who answered 1 “it occurs very rarely” (rare) and 5 “it occurs very often” (often)*.

c Low control is represented by the percentage of participants who answered “it is very difficult to cease the experience” and “it is very difficult to avoid the experience,” whereas high control is represented by the percentage of participants who answered “it is very easy to cease the experience” and “it is very easy to avoid the experience.”

d *Percentage of participants who answered “the experience is very negative” (negative) and “the experience is very positive” (positive)*.

Independent samples *t*-tests revealed that neither the LSHS total scores nor the factor score for all the phenomenological characteristics of the hallucinatory experiences (prevalence, frequency of occurrence, degree of control, and affective content) were significantly different as a function of participant's sex (*p* > 0.50 for all cases).

Of note, the prevalence of hallucinatory experiences was not correlated with the use of alcohol or drugs (*r* = 0.04, *p* = 0.50).

Table 6 illustrates the overall prevalence, frequency of occurrence, and perceived degree of control for each item of the LSHS. In general, prevalence of hallucinatory experiences reported by the participants ranged between a minimum of 5% for item 9 and a maximum of 70% for item 3. Moreover, experiences described in items 1, 2, 3, 7, and 12 received the highest prevalence and frequency of occurrence ratings, whereas experiences described in items 9, 10, and 16 were the least prevalent and frequent. Experiences described in items 1, 2, 3, 7, and 12 were perceived as the most difficult to control.

**Table 6:** Overall number of participants and (percentages) for each of the 16 LSHS items according to prevalence, frequency of occurrence, and perceived degree of control.

**Item**	**Prevalence^a^**	**Frequency^b^ (rare-often)**	**Control^c^ (low-high)**
1.	146 (41%)	5–118 (1–33%)	137–3 (39–0.8%)
2.	172 (49%)	3–139 (0.8–39%)	162–3 (46–0.8%)
3.	249 (70%)	1–206 (0.3–58%)	233–8 (66–2%)
4.	65 (18%)	13–45 (4–13%)	57–6 (16–2%)
5.	90 (25%)	4–73 (1–21%)	81–4 (23–1%)
6.	67 (19%)	1–62 (0.3–17%)	63–0 (18–0%)
7.	119 (34%)	2–93 (0.6–26%)	107–5 (30–1%)
8.	53 (15%)	1–48 (0.3–14%)	52–1 (15–0.3%)
9.	19 (5%)	4–13 (1–4%)	18–1 (5–0.3%)
10.	20 (6%)	3–13 (0.8–4%)	18–1 (5–0.3%)
11.	79 (22%)	14–53 (4–15%)	73–3 (21–0.8%)
12.	192 (54%)	22–141 (6–40%)	171–12 (48–3%)
13.	52 (15%)	7–39 (2–11%)	49–2 (14–0.6%)
14.	40 (11%)	5–29 (1–8%)	37–1 (10–0.3%)
15.	36 (10%)	4–29 (1–8%)	34–0 (10–0%)
16.	22 (6%)	2–16 (0.6–4%)	20–1 (6–0.3%)

a *Percentage of participants who answered “possibly applies to me” or “definitely applies to me” (3 or 4 points)*.

b *Percentage of participants who answered 1 “it occurs very rarely” (rare) and 5 “it occurs very often” (often)*.

c Low control is represented by the percentage of participants who answered “it is very difficult to cease the experience” and “it is very difficult to avoid the experience,” whereas high control is represented by the percentage of participants who answered “it is very easy to cease the experience” and “it is very easy to avoid the experience.”

Considering the items associated with Factor I (items 4, 8, 9, 10, 14, 15, and 16), auditory hallucinations (13%) were reported as more prevalent than olfactory (11%), tactile (10%), and visual hallucinations (6%). The highest ratings of frequency and low control for the four types of hallucinations (Factor I) were also observed in the case of auditory hallucinations (10 and 12%, respectively), followed by olfactory and tactile hallucinations (8% for both and 10% for both, respectively), and visual hallucinations (4 and 5%, respectively).

#### Other phenomenological features

Overall, a great number of participants described their experiences as concerning them personally (65%), involving relatives or friends (41%), and personally experienced events (38%). Hallucinatory experiences related to previous experiences occurred recently in 16% of participants, between 1 and 5 years ago in 15% of participants, and more than 5 years ago in 7% of participants. Moreover, 42% of participants reported that their experiences occurred during stressful events or difficulties. Finally, a small percentage of participants (5%) described their hallucinations as being experienced under the influence of alcohol or drugs.

#### Relationship between the affective content of the hallucinatory experiences, perceived degree of control, and frequency of occurrence

**Figure 1:**
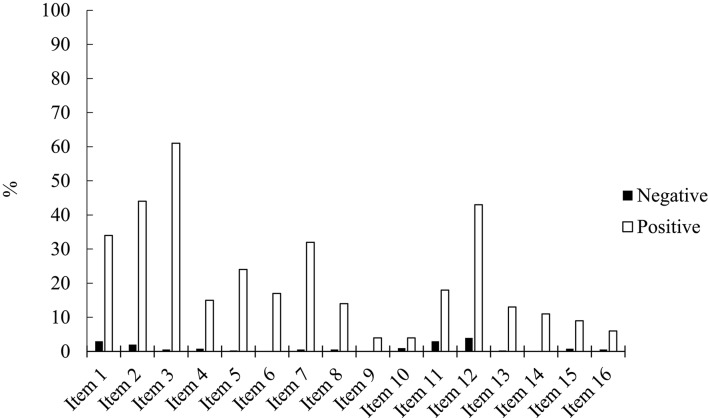
Percentage of negative and positive affective ratings for each LSHS item.

Figure 1 summarizes the percentage of participants who rated their experiences as being pleasant vs. unpleasant. All experiences were perceived as more pleasant than unpleasant. Pleasant ratings ranged between a minimum of 4% for items 9 and 10, and a maximum of 61% for item 3, whereas unpleasant ratings ranged between a minimum of 0% for items 6, 9, and 14, and a maximum of 4% for item 12.

The perceived degree of control, both in terms of ceasing the experience (*r* = 0.53, *p* < 0.001) and preventing the reappearance of the experience (*r* = 0.60, *p* < 0.001), was significantly correlated with the affective content of the hallucinatory experiences: the more pleasant the experiences were described, the more controllable they were perceived. A strong correlation between the affective content of the hallucinatory experiences and the frequency of occurrence was also found (*r* = 0.62, *p* < 0.001), which suggests that these experiences tend to be described as more pleasant as more often they occur.

### Relationship between LSHS scores and clinical scores (anxious-depressive symptomatology and schizotypal tendencies)

Clinical scores differed significantly between the two subsamples (see Table 7), with individuals with high hallucination predisposition showing higher scores than individuals with low hallucination predisposition in both clinical measures (BSI and SPQ).

**Table 7:** Mean values and standard deviations of the LSHS, BSI, and SPQ considering higher hallucination predisposition and lower hallucination predisposition subsamples separately.

**Clinical measures**	**Higher hallucination predisposition (*****n*** = **16)**		**Lower hallucination predisposition (*****n*** = **14)**		
	***M***	***SD***	***M***	***SD***	***t***
LSHS Total score	35.13	8.03	14.57	4.69	−8.69^***^
BSI Somatization	2.85	2.73	1.20	1.20	−2.20^*^
BSI Obsessive-compulsive	4.50	2.75	2.35	1.82	−2.54^*^
BSI Interpersonal sensitivity	1.80	1.40	0.93	0.93	−2.05^*^
BSI Depression	3.81	1.94	1.65	1.10	−3.80^**^
BSI Anxiety	4.01	2.35	2.00	1.66	−2.80^**^
BSI Hostility	3.20	2.15	1.80	1.64	−2.06^*^
BSI Phobic anxiety	1.20	1.30	0.54	0.90	−1.90^+^
BSI Paranoid ideation	2.95	2.40	1.91	1.80	−1.40
BSI Psychoticism	1.75	1.91	0.63	0.84	−2.12^*^
BSI Positive symptoms distress index	1.46	0.31	1.26	0.27	−1.90^+^
SPQ Cognitive-perceptual factor	11.00	4.83	6.93	4.10	−2.50^*^
SPQ Interpersonal factor	11.69	5.20	7.60	3.82	−2.50^*^
SPQ Disorganized factor	4.94	3.04	2.60	1.55	−2.73^*^
SPQ Total score	24.31	7.14	14.71	6.10	−3.98^***^

*** *p* < *0.001*;

** *p* < *0.010*;

* *p* < *0.050*;

+ *p* < *0.10*.

Moreover, the prevalence of hallucinatory experiences was strongly correlated with clinical symptomatology (BSI Obsessive-Compulsive, *r* = 0.46, *p* = 0.011; BSI Depression, *r* = 0.43, *p* = 0.017; BSI Anxiety, *r* = 0.45, *p* = 0.012; BSI Hostility, *r* = 0.41, *p* = 0.024; BSI Positive Symptom Distress Index, *r* = 0.50, *p* = 0.005) and schizotypal tendencies (SPQ Total Score; *r* = 0.43, *p* = 0.016). Participants who reported having more hallucinations presented increased anxiety and depressive symptoms, as well as schizotypal tendencies.

## Discussion

The current study aimed to translate, adapt and validate the 16-item Launay-Slade Hallucinations Scale (Larøi and van der Linden, 2005b) for the Portuguese population. Further, the phenomenology of hallucinations was explored in a sample of European Portuguese college students, as well as the relationship between hallucinatory experiences and the use of alcohol and drugs, following Larøi and van der Linden (2005b). An additional goal was to probe the relationship between hallucinatory experiences and anxious-depressive symptoms and schizotypal traits.

### The dimensional structure of the portuguese LSHS

The results demonstrate that the Portuguese adaptation of the LSHS is characterized by high internal validity, as well as by high internal consistency and reliability. The exploratory factor analysis showed that a three-factor structure is the most adequate solution for the LSHS Portuguese version (Factor I: “Auditory, visual, olfactory, and tactile hallucinations,” Factor II: “Vividness of daydreams,” and Factor III: “Intrusive or vivid thoughts and Sleep-related hallucinations”). The original five-factor solution proposed by Larøi and van der Linden (2005b), as well as the subsequent Italian four-factor solution (Vellante et al., 2012), did not meet the standards of fit in the current study. Our results provide further evidence for the multifactorial structure of hallucination predisposition measured by the LSHS (e.g., Levitan et al., 1996; Morrison et al., 2000; Larøi et al., 2004; Serper et al., 2005; Paulik et al., 2006; Fonseca-Pedrero et al., 2010; Vellante et al., 2012), even though the number of factors that better represent these experiences vary consistently across studies (e.g., Aleman et al., 2001; Waters et al., 2003; Larøi et al., 2004; Larøi and van der Linden, 2005b; Serper et al., 2005; Vellante et al., 2012). As most of these studies have used shorter versions of the LSHS (e.g., Launay and Slade, 1981; Bentall and Slade, 1985; Morrison et al., 2000, 2002; Aleman et al., 2001; Serper et al., 2005), a comparison of the current results was only possible with the studies of both Larøi and van der Linden (2005b) and Vellante et al. (2012).

Important discrepancies between the Belgian validation and our study were observed. First, in the current study auditory and visual hallucinations were combined into a single factor (Factor I), whereas intrusive or vivid thoughts and sleep-related hallucinations were combined as one factor (Factor III). Each of these experiences (auditory hallucinations, visual hallucinations, intrusive or vivid thoughts and sleep-related hallucinations) emerged as four separate factors in the original study of Larøi and van der Linden (2005b; Factor IV, Factor V, Factor III, and Factor I, respectively). Nevertheless, we replicated the combination of auditory and visual hallucinations into a single factor, previously reported in the Italian adaptation of this LSHS version (Vellante et al., 2012). This suggests that hallucinatory experiences in the auditory and visual modalities share phenomenological similarities (e.g., automatic nature, occurrence during waking hours). On the other hand, intrusive thoughts have been found to be associated with hypnagogic or hypnopompic hallucinations (Schmidt and Gendolla, 2008; Jones et al., 2009; McCarthy-Jones et al., 2011), which might explain why both hallucinatory experiences emerged as a single factor (Factor III) in the current study.

Second, olfactory and tactile sensory modalities of hallucinations were also combined with auditory and visual hallucinations in the current study, resulting in a multisensory factor. This combination is in contrast with Larøi and van der Linden (2005b): whereas the item concerning olfactory hallucinations (item 14) was excluded, the tactile hallucinations item (item 15) was part of Factor I (“Sleep-related hallucinations”). Tactile hallucinations are most likely to occur while falling asleep (hypnagogic experiences) or waking up (hypnopompic experiences; Ohayon, 2000; Cheyne, 2001). However, they can also be experienced during waking hours (e.g., Tien, 1991). In the current study, the combination of four sensory modalities of hallucinations (auditory, visual, olfactory, and tactile) into a single factor (Factor I “Auditory, visual, olfactory, and tactile hallucinations”) is consistent with the observation that all these experiences occur in a state of complete awareness (wakefulness hallucinations; e.g., Tien, 1991; Ohayon, 2000).

A similar finding between the current and the original study (Larøi and van der Linden, 2005b) was only evident in Factor II (“Vividness of daydreams”), as it includes the same items indicated by Larøi and van der Linden (2005b). This suggests that experiences reported in Factor II might be more easily differentiated from other forms of hallucinations. As for the internal consistency and reliability of the questionnaire, the analysis yielded satisfactory results. The current indices (alpha coefficient and item-total correlations) reached similar values to those described in the original study, suggesting a high reliability of the LSHS Portuguese version.

### Phenomenological aspects of hallucinations in portuguese nonclinical individuals

Overall, 10% of the sample reported significant hallucinatory experiences (LSHS score > 35). This finding fits well with previous observations reporting an incidence of 10–15% of hallucinators with no clinical diagnosis (e.g., Barrett and Etheridge, 1992; Paulik et al., 2006; Badcock et al., 2008; Sommer et al., 2010). In the current sample, the experiences were neither under the influence of alcohol and drugs, as only 5% of participants described their hallucinations as being experienced under the influence of alcohol or drugs, and occurred similarly in male and female participants. The experience of hallucinations was associated with stressful events (42%), in line with previous studies (e.g., Moskowitz and Corstens, 2008; Crowe et al., 2011; Larøi, 2012). Even though in a small proportion of cases (24%), hallucination predisposition was also associated with stressful events in the study of Larøi and van der Linden (2005b). Despite variability in the prevalence and frequency ratings of hallucinatory experiences in the current sample, intrusive or vivid thoughts and hypnagogic and hypnopompic hallucinations (i.e., experiences occurring between waking and sleeping or during sates of altered awareness) were the most common (42%) and frequent (33%), whereas wakefulness hallucinations (i.e., experiences occurring during waking hours or states of complete awareness) were the least prevalent (10%) and frequent (8%). A closer inspection of each item of Factor I (“Auditory, visual, olfactory, and tactile hallucinations”) indicated that auditory hallucinations were relatively common in the Portuguese sample (18% for item 4; 15% for item 8), although not as common as in the Belgian sample (Larøi and van der Linden, 2005b; 34 and 19%, respectively). At the same time, auditory hallucinations were a less prevalent experience in the current sample (13%) than in previous reports with clinical groups (~70% in schizophrenia; 20–50% in bipolar disorder; and 40% in posttraumatic stress disorder; Choong et al., 2007). Consistent with this finding, epidemiological studies suggest that patients are more likely to report auditory hallucinations than nonpsychotic individuals (e.g., Tien, 1991; Johns et al., 2004; de Leede-Smith and Barkus, 2013). Moreover, the presence of hypnagogic and hypnopompic hallucinations is higher in nonclinical individuals than in patients (de Leede-Smith and Barkus, 2013), whereas patients experience wakefulness hallucinations more often than nonclinical individuals (Ohayon, 2000; Cheyne, 2001; Jones et al., 2009).

As visual hallucinations tend to be the most common form of wakefulness hallucinations in nonclinical groups (Tien, 1991; Ohayon, 2000; Larøi and van der Linden, 2005b; Simon et al., 2009; Sommer et al., 2010), we did not expect to observe that auditory hallucinations (13%) were more prevalent than visual hallucinations (6%). Interestingly, olfactory (11%) and tactile (10%) hallucinations were also more prevalent when compared with visual hallucinations. Consistent with previous studies, differences between sensory modalities of hallucinations in nonclinical individuals were minimal (Tien, 1991; Johns and van Os, 2001). These results corroborate the multimodal nature of nonclinical hallucinations (e.g., Tien, 1991; Johns et al., 2004; de Leede-Smith and Barkus, 2013).

Regarding the perceived control over the hallucinations, all types of hallucinatory experiences were rated as highly uncontrollable and dominating. Specifically, intrusive or vivid thoughts and hypnagogic and hypnopompic hallucinations were perceived as uncontrollable experiences by a greater number of participants (39%). Furthermore, auditory hallucinations were perceived as more difficult to control (12%) than olfactory (10%), tactile (10%), and visual (5.5%) hallucinations. This finding agrees with previous studies (Larøi and van der Linden, 2005b), and additionally suggests that the inability to control such experiences might be comparable to that described by clinical individuals (Chadwick and Birchwood, 1994).

Regarding the emotional content of hallucinatory experiences, wakefulness hallucinations were predominantly perceived as pleasant experiences by Portuguese participants (9%), whereas they were perceived more frequently as unpleasant by Belgian participants (34%). In addition, intrusive or vivid thoughts and hypnagogic and hypnopompic hallucinations were also perceived as more pleasant (35%) than the other hallucinatory experiences in the current study. Also, only 0.7% of participants perceived auditory hallucinations as unpleasant in this study. Nevertheless, auditory hallucinations were also rated as positive experiences by a significant number of participants (23%) in the study of Larøi and van der Linden (2005b). In line with these results, prior studies demonstrated that positive emotional reactions to hallucinatory experiences tend to be more common among nonclinical individuals (de Leede-Smith and Barkus, 2013), whereas the opposite is observed in psychotic patients (e.g., de Leede-Smith and Barkus, 2013; Pinheiro et al., 2016).

As expected, the perceived emotional content of hallucinatory experiences and the degree of control over those experiences were associated. Specifically, the more pleasant the hallucinatory experiences were perceived, the more controllable they were judged. A relationship between both variables was also found in the original study (Larøi and van der Linden, 2005b), but in the opposite direction (i.e., unpleasant experiences were associated with a lower degree of control). Nevertheless, it is reasonable to expect that pleasant experiences are more easily controllable than unpleasant experiences. Consistent with Larøi and van der Linden (2005b), hallucinatory experiences were also described as more pleasant as more often they tend to occur. The perceived pleasantness may result from the increased familiarity associated with frequent hallucinatory experiences (Nayani and David, 1996; Larøi and van der Linden, 2005b). In sum, the current findings corroborate previous observations of less severe hallucinatory experiences in nonclinical compared to clinical individuals (e.g., de Leede-Smith and Barkus, 2013). Note that these experiences were, at the same time, less unpleasant than those reported in Larøi and van der Linden (2005b), which lends support to the existence of a continuum of severity even in the nonclinical experience of hallucinations (Taylor et al., 2016).

### Relationship between hallucination predisposition and clinical symptoms

Individuals with higher hallucination predisposition presented more anxious-depressive and schizotypal symptomatology. This result fits well with evidence showing that healthy individuals who experience hallucinations may share psychophysiological similarities with psychotic patients, including feelings of anxiety and depression (Bentall et al., 1989; Yung et al., 2006, 2009; de Leede-Smith and Barkus, 2013). Moreover, the current findings add support to the observation that the presence of both anxious-depressive symptomatology and schizotypal tendencies is related to hallucinatory experiences (Tien and Eaton, 1992; Yung and McGorry, 1996; Paulik et al., 2006; Smith et al., 2006; Barkus et al., 2007; de Leede-Smith and Barkus, 2013). However, the direction of the association needs further examination in future studies: individuals with increased psychopathological symptoms may be more vulnerable to hallucinatory experiences, or vice-versa. Importantly, the combination of psychotic symptoms (e.g., hallucinations and schizotypal tendencies) and negative mood (e.g., anxiety and depression) may place individuals at more risk for a subsequent conversion to psychosis (e.g., Jones et al., 1994; Yung et al., 2006; Armando et al., 2010; de Leede-Smith and Barkus, 2013). This is particularly evident in schizophrenia, in which symptoms of depression (e.g., Leff et al., 1988) and anxiety (e.g., Cosoff and Hafner, 1998; Turnbull and Bebbington, 2001) are frequent comorbidities.

## Limitations

Some limitations should be considered when interpreting the current findings. First, the sample is comprised of college students who were motivated to participate in the study, and hence it is not representative of the general (nonclinical) population. Second, the factor structure of the LSHS Portuguese version was obtained only via exploratory factor analyses, and not confirmed by confirmatory factor analyses. Finally, regarding the relationship between the hallucinatory experiences and psychopathological and schizotypal tendencies, the observed association results from a correlational analysis, which does not allow inferences about causality.

## Conclusion

The current study shed further light on hallucinatory experiences in the nonclinical population. Portuguese nonclinical individuals were not only more likely to experience intrusive or vivid thoughts and hypnagogic and hypnopompic hallucinations relative to the other five types of hallucinations (auditory, visual, olfactory, tactile, and vividness of daydreams hallucinations), but also to perceive their hallucinatory experiences as less unpleasant than what is commonly reported in individuals with a psychotic disorder. Of note, the current results demonstrate that hallucination predisposition is related to clinical symptomatology (schizotypal tendencies and negative mood), which may represent increased psychotic risk.

The psychometric proprieties of the LSHS here examined suggest that this scale is a reliable and valid questionnaire to objectively assess hallucinatory experiences in the nonclinical population. Therefore, the adaptation of the LSHS for the Portuguese population represents a useful tool not only for further research intended to clarify the phenomenology of hallucinations in nonclinical individuals, but also for comparative research probing the effects of culture on hallucinatory experiences.

## Author contributions

AP conceived and designed the study and supervised data collection. PC collected, analyzed and interpreted the data, and produced the drafting of the manuscript. AP supervised all steps in the study and provided a critical revision of the manuscript.

### Conflict of interest statement

The authors declare that the research was conducted in the absence of any commercial or financial relationships that could be construed as a potential conflict of interest. The handling Editor declared a shared affiliation, though no other collaboration, with one of the authors AP and states that the process nevertheless met the standards of a fair and objective review.

## Appendix A

**Table A1:** Portuguese version of the Launay-Slade Hallucinations Scale.

1.	Por vezes, um pensamento passageiro parece-me tão real que me assusta.
2.	Por vezes, os meus pensamentos parecem tão reais como as coisas que acontecem de verdade.
3.	Por muito que tente concentrar-me, acabam sempre por me vir à mente pensamentos que não estão relacionados com aquilo que estou a fazer.
4.	No passado, tive a experiência de ouvir a voz de uma pessoa, tendo-me apercebido, de seguida, que afinal não havia ali ninguém.
5.	Os sons que ouço quando sonho acordado(a) são, geralmente, claros e nítidos.
6.	As pessoas que aparecem nos meus sonhos, quando sonho acordado(a), parecem tão reais que, por vezes, penso mesmo que existem.
7.	Quando sonho acordado(a), consigo ouvir o som de uma melodia quase tão nitidamente como se estivesse realmente a ouvi-la.
8.	Ouço frequentemente uma voz que diz os meus pensamentos em voz alta.
9.	Já me senti incomodado(a) por ouvir vozes na minha cabeça.
10.	Em certas ocasiões, vi o rosto de uma pessoa em frente a mim quando, na realidade, não estava ali ninguém.
11.	Por vezes, imediatamente antes de adormecer ou ao acordar, tive a experiência de ver, sentir ou ouvir algo ou alguém que não estava presente, ou a sensação de ser tocado apesar de ninguém estar presente.
12.	Por vezes, imediatamente antes de adormecer ou ao acordar, tive a sensação de flutuar, ou de cair, ou de abandonar o meu corpo temporariamente.
13.	Em certas ocasiões, tive a sensação de presença de alguém próximo que já faleceu.
14.	No passado, experienciei um odor particular apesar de este não existir.
15.	Já tive o sentimento de tocar algo ou de ser tocado(a), apesar de não haver nada ou ninguém por perto.
16.	Por vezes, vi coisas ou animais quando na realidade não havia nada ali.
